# Harnessing Biomaterials for Immunomodulatory-Driven Tissue Engineering

**DOI:** 10.1007/s40883-022-00279-6

**Published:** 2022-09-29

**Authors:** Justin X. Zhong, Preethi Raghavan, Tejal A. Desai

**Affiliations:** 1 UC Berkeley – UCSF Graduate Program in Bioengineering, San Francisco, CA 94143 USA; 2 Department of Bioengineering and Therapeutic Sciences, University of California, San Francisco, CA 94143 USA; 3 Department of Bioengineering, University of California, Berkeley, CA 94720 USA; 4 School of Engineering, Brown University, Providence, RI 02912 USA

**Keywords:** Biomaterials, Immunomodulation, Tissue engineering, Regenerative medicine, Drug delivery

## Abstract

**Abstract:**

The immune system plays a crucial role during tissue repair and wound healing processes. Biomaterials have been leveraged to assist in this in situ tissue regeneration process to dampen the foreign body response by evading or suppressing the immune system. An emerging paradigm within regenerative medicine is to use biomaterials to influence the immune system and create a pro-reparative microenvironment to instigate endogenously driven tissue repair. In this review, we discuss recent studies that focus on immunomodulation of innate and adaptive immune cells for tissue engineering applications through four biomaterial-based mechanisms of action: biophysical cues, chemical modifications, drug delivery, and sequestration. These materials enable augmented regeneration in various contexts, including vascularization, bone repair, wound healing, and autoimmune regulation. While further understanding of immune-material interactions is needed to design the next generation of immunomodulatory biomaterials, these materials have already demonstrated great promise for regenerative medicine.

**Lay Summary:**

The immune system plays an important role in tissue repair. Many biomaterial strategies have been used to promote tissue repair, and recent work in this area has looked into the possibility of doing repair by tuning. Thus, we examined the literature for recent works showcasing the efficacy of these approaches in animal models of injuries. In these studies, we found that biomaterials successfully tuned the immune response and improved the repair of various tissues. This highlights the promise of immune-modulating material strategies to improve tissue repair.

## Introduction

Regeneration and repair have been the long-term goals of the field of tissue engineering [1]. As the field expands its understanding of the biology surrounding tissue engineering and repair, it has become abundantly clear that the immune system plays a critical role in facilitating these processes [2, 3]. Within canonical wound healing processes, immune cells are present at every step. Therefore, dictating how the immune system responds to injuries and disease will be key to promoting endogenous repair.

It has been long known that biomaterials interact with the immune system, notably in the resulting host reaction from implanted materials, leading to a foreign body response [4]. Many studies have explored biomaterial strategies to modulate the immune system to mitigate and overcome the foreign body response [5, 6]. Immunomodulatory biomaterials have also been leveraged for other applications, notably cancer therapeutics [7, 8], and vaccines [9, 10]. More recently in the last decade, a new paradigm has emerged in regenerative medicine to leverage immunomodulatory biomaterials for tissue engineering applications [11–14]. Biomaterials have been used within the context of tissue engineering for over 20 years; however, this new approach seeks to utilize biomaterial platforms to influence endogenous immune cell populations and create pro-reparative microenvironments [15, 16].

**Fig. 1:**
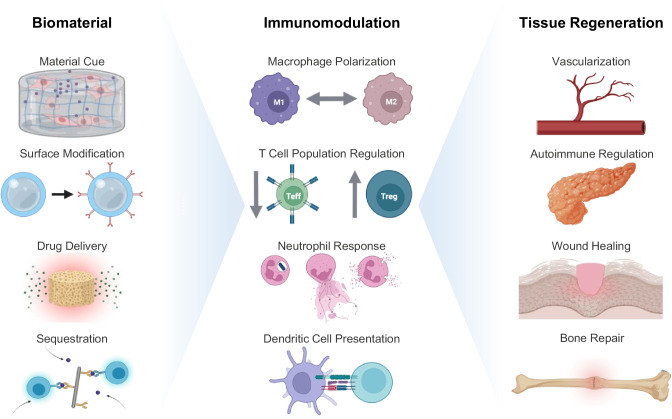
Biomaterials can modulate the immune system for tissue regenerative purposes through four main categories: biophysical cues, chemical modification, drug delivery, or sequestration

In this review, we will provide a brief overview of immune cell populations and how material cues have been used to control their responses. We will then examine studies exploring tissue engineering applications with immunomodulatory biomaterials from the last 5 years, where we identify four mechanisms of action utilized in the design of these materials: biophysical cues, chemical modifications, drug delivery, and sequestration of endogenous factors (Fig. 1). We discuss how different biomaterial platforms exert changes on the immune response to encourage tissue repair and regeneration and offer perspectives on the future direction of the field.

## Tuning Immune Cell Behaviors Through Material Cues

Following tissue injury, damaged tissue undergoes four stages of repair in canonical wound healing: hemostasis, inflammation, proliferation, and remodeling [3]. Throughout this process, immune cells interface with parenchymal, stromal, and other immune cells to advance the tissue through each stage by clearing cellular debris, secreting biochemical cues, and dictating the formation of the new microenvironment. Immune cells will appear transiently throughout the various stages, with innate immune cells appearing in early stages and adaptive immune populations aiding later stages [17]. Disruption and dysregulation of immune function through trauma or disease, as discussed elsewhere [18–22], can lead to persistent activation of inflammatory or anti-inflammatory states and subsequently complications in repair, as seen in autoimmune diseases and fibrosis. Implantation of foreign materials are met with similar responses, but presentation of physical and biochemical cues by these materials can mediate and skew immune signaling. In this section, we will briefly describe the various immune cells, their role in the repair process, and summarize some of their divergent responses to material cues.

### Innate Immune System

Macrophages play a key role in the innate immune system’s response to wounds, to inflammatory cues, clearing pathogens, and cellular debris, and mediating the downstream remodeling process through cellular cross-talk. In response to local stimuli, macrophages activate to perform different subsets of functions. These activation states exist on a multi-axial spectrum, where different signals induce specific transcriptional responses [23]. Early within the wound healing process, macrophages tend to exhibit a pro-inflammatory “M1-like” state to recruit other immune cells and drive the inflammation process. Macrophages then switch to a pro-regenerative “M2-like” to resolve inflammatory microenvironments and promote tissue repair. The role macrophages play throughout this process is also impacted by the origin of the macrophage, as tissue-resident macrophages have been shown to be more reparative in comparison to their circulating counterparts [24, 25]. To this end, driving macrophages away from inflammatory states and towards anti-inflammatory, anti-fibrotic, and tissue repair phenotypes have been shown to be beneficial for regeneration [26–28]. The effect of material cues and biomaterial design to drive these phenotypic shifts has been discussed in depth in other reviews, but it is clear that various materials and their properties can impact macrophage polarization [29–34]. Understanding how different materials will impact macrophage behavior is key for rational design and selection. Synthetic and biological materials elicit different macrophage immune responses [35], with a more fibrotic response towards synthetic materials whereas biological materials elicited a type-2 response that improved repair. Further examination of the resulting macrophage populations reveals heterogeneous macrophage phenotypes important in the repair and fibrosis processes [36]. There is a need for a variety of macrophage states during the tissue remodeling process, as inflammatory and pro-reparative phenotypes are often needed in sequence to drive vascularization and angiogenesis [37, 38]. Failure to transition from inflammatory to reparative states can inhibit successful tissue repair [39, 40]. However, the molecular axes by which biomaterials elicit phenotypic changes is not always well understood and remains an area of ongoing investigation in the field. Deciphering the immunological response to material therapeutics provides further guidelines for material design and highlights novel population subsets to target. To tune macrophage responses to materials, our lab and others have shown that the addition of micro and nanotopographical cues on materials can mediate inflammatory cytokine production and guide macrophage activation and activities [41–44]. Furthermore, the stiffness and molecular weight of material cues have also demonstrated differential activation of macrophages. Increasing the stiffness of polyacrylamide gels led to increased pro-inflammatory gene expression and migration behaviors [45]. As a soluble cue, increasing the molecular weight of hyaluronic acid promoted anti-inflammatory gene expression in macrophages, while shorter chain lengths led to increased inflammatory expression [46].

Neutrophils are one of the first innate immune cells to respond to infection and inflammation, responding to stimulation to undergo one of three different functions: degranulation, apoptosis, and the formation of neutrophil extracellular traps (NETosis). All these functions can result in increased levels of neutrophil-derived antibacterial proteins and proteases, which can cause further inflammation and recruitment of other immune cells in the tissue [47]. The biomaterial-neutrophil interface for tissue engineering applications has not been extensively investigated. The short lifespan of neutrophils in circulation coupled with its complex biological regulation have hindered advancements in its applications in tissue engineering and biomaterials. Preliminary research has focused on characterizing neutrophil function at the biomaterial interface, such as apoptosis or NETosis, particularly in the context of the foreign body response to implanted devices [48–50].

other innate immune cells include dendritic cells, natural killer cells, basophils, eosinophils, and mast cells. These cell populations play roles in wound healing by aiding in the resolution of inflammation and beginning repair processes in the proliferation phase [51–53]. Dendritic cells (DCs) play an important role in connecting the innate and adaptive immune cell by presenting various stimulatory signals to adaptive immune cells, such as T cells. In the context of wound healing and tissue repair, DCs can direct the adaptive immune system response either to drive inflammation or regeneration in response to a biomaterial [54]. While this field is still relatively unexplored, a growing focus of DC-targeting biomaterials is to create vaccines and vaccine adjuvants [55, 56]. How these innate immune populations respond to material cues have not been well studied yet, as current work has focused on better understanding cellular behavior in vitro [57]. Understanding how to tune cellular responses to biomaterial-cell interfaces through modulation of material properties is essential to developing material-based tissue engineering approaches to influence tissue repair through these axes of the innate immune system.

### Adaptive Immune System

The adaptive immune system plays a key role in reparative processes, exerting effects most prominently in the proliferation and remodeling phases. T cells respond to a host of cues from the innate immune system, making them central to the adaptive immune response as they produce cytokines and regulate the immune system. They can broadly be categorized as either CD4^+^ helper cells or CD8^+^ cytotoxic cells and exhibit a variety of subsets, each with their own functions. While high levels of CD8^+^ cytotoxic T cells have been shown to impede wound repair, different subsets of CD4^+^ helper T cells are beneficial for specific immune responses [17, 58]. In addition to effector CD4^+^ helper T cell subsets, CD4^+^ regulatory T cells (Tregs) can encourage immunosuppression that leads to homeostasis and improved wound healing. It has been shown that biomaterials that skew T cell responses towards a protective Th2 response promote a regenerative phenotype, reiterating the importance of rational material selection for regenerative engineering applications [59]. Furthermore, the design of materials to activate these various T cell subtypes has been thoroughly investigated, where parameters including micro and nanostructures, material dynamics, and signal presentation all play key roles in dictating activation [60, 61]. Topographical cues on material surfaces influence the immune synapse organization on T cell surfaces, leading to differential cytokine release in a mechanosensitive manner [62]. Many materials seek to dictate T cell activation through mimicry of the immune synapse, acting as artificial antigen presenting cells through replication of activation signals. On these materials, the ligands presented as well as the spatial and ratiometric presentation of signals control the subtypes of T cells activated and their subsequent responses [63–66]. Beyond manipulating the immune synapse, the molecular weight of hyaluronan has demonstrated specific upregulation of transcription factors for maintenance of Treg phenotypes [67].

B cells play a key role in the adaptive immune system through their production of antibodies in response to antigens, as well as their secretion of cytokines. To this end, however, the role of B cells in the context of repair has been much less studied [17], as both the presence and absence of B cells have demonstrated positive influences on wound healing [68, 69]. These divergent responses have also been observed in response to material implants, where material cues influence B cell antigen presentation and inflammatory gene expression to promote more fibrotic or reparative responses [70]. While material modulation of B cell responses has largely been focused on the development of vaccines and cancer therapies [7, 71, 72], further exploration is needed in the design of materials to create immuno-reparative microenvironments through B cell modulation.

## Material-Directed Immunological-Based Tissue Remodeling

Biomaterials can interface with the local microenvironment to influence the immune system through a variety of methods. Here, we describe recent advancements in tissue engineering applications within the field of immunomodulatory biomaterials through the biomaterial’s mechanisms of action, including: biophysical cues, chemical modifications, drug delivery, and sequestration (Table 1).

**Table 1:** Summary of immunomodulatory biomaterial strategies

		Target Immune Populations for Modulation	
		Innate Immune	Adaptive Immune
Biomaterial Mechanism of Action	Biophysical Cues	Refs. [76, 79, 80, 84, 85]	Refs. [81, 82]
	Chemical Modifications	Refs. [84–86, 88–92]	Refs. [94–97]
	Drug Delivery	Refs. [101–105, 107]	Refs. [106, 108–110, 113]
	Sequestration	Refs. [121]	Refs. [118, 126]

### Biophysical Cues

The inherent material properties of a platform can influence the local innate and adaptive immune response. This can include mechanical characteristics of the biomaterial such as the shape, dimensionality, micro- and nano-topography, and stiffness. Several reviews have covered the effect of these material cues on various cell functions such as migration, adhesion, proliferation, and differentiation in a variety of biological contexts [73, 74]. These mechanical and physical properties can alter immune cell phenotypes and dictate if the local microenvironment is inflammatory or reparative. The ability for biophysical properties of a material alone to drive immunomodulation demonstrates promise for acellular tissue engineering platforms moving forward.

#### Biophysical Cues: Innate Immune Response

The effect of biomaterial topography on the innate immune system is of great interest, as changes in topography can alter macrophage shape, and therefore downstream macrophage polarization. Zhu et al. recently found that by modifying the scale of honeycomb structures in titanium dioxide they could control the level of macrophage polarization. Smaller structures resulted in M2 polarization and helped drive integration of bone implants [75]. For electrospun materials, physical properties such as template material and diameter of fibers can influence immune response. Neutrophils are less studied in the context of wound healing, but researchers found that altering electrospun fiber properties could mitigate levels of NETosis and contribute to enhanced tissue integration and regeneration [76]. High levels of NETosis can perpetuate an inflammatory environment and lead to downstream recruitment of other innate and adaptive immune cells, therefore further research in this area could greatly advance the field of immunomodulatory biomaterials.

**Fig. 2:**
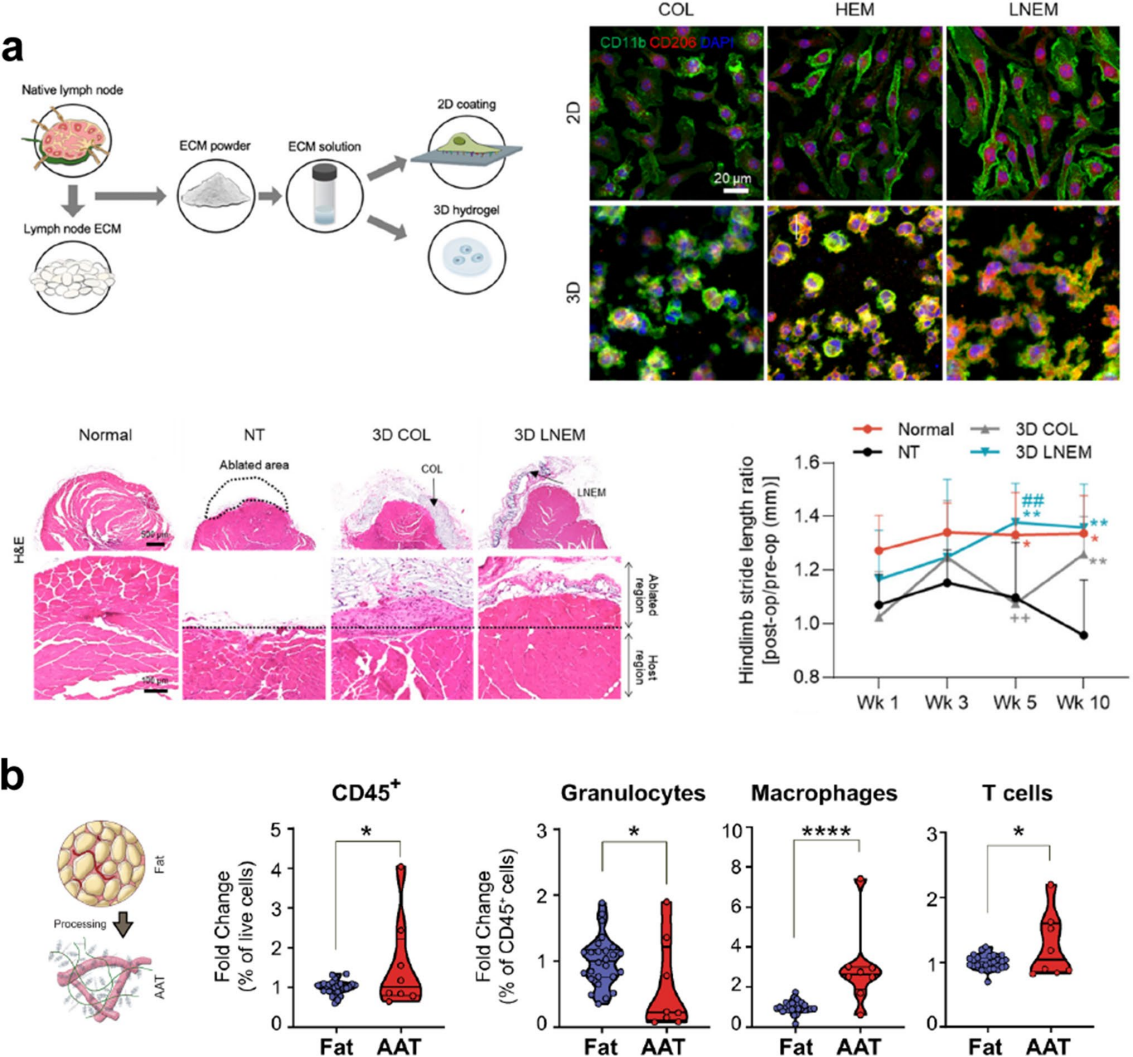
Biophysical cues. (**a**) A three-dimensional lymph node derived extracellular matrix led to macrophage elongation, enhanced M2 expression and secretion of anti-inflammatory cytokines. Pan-macrophage marker CD11b and M2 marker CD206 show highest expression of M2 in the 3D LN-ECM. After implantation in a mouse model of volumetric muscle loss, H&E shows improved muscle regeneration in the 3D LN-ECM, lowered levels of fibrosis and adipogenesis, and improved gait. Adapted from Ref. [79]. (**b**) An adipose-derived ECM for soft tissue reconstruction was tested in Phase I clinical trials. They show that within implants excised from there is an influx of immune cells (CD45.+) within the implant — specifically lower granulocytes, higher M2 macrophages, and higher regulatory T cells. Adapted from Ref. [81]

Extracellular matrices (ECM) provide native architecture to help guide tissue repair following injury. Compositionally, the network of proteins and glycosaminoglycans provides physical and chemical cues for mediation of cellular phenotypes. Furthermore, native ECM also can both release embedded cytokines and sequester endogenous cytokines to dictate regeneration within the local microenvironment [77]. Ultimately, harnessing ECM-based materials for exogenous applications seeks to recapitulate all of these functions, yet current processes for extraction and decellularization prohibit complete functionality [78]. Many papers focus on investigating the effect of decellularized ECM on the innate immune system; Choi et al. recently looked at how specifically dimensionality of decellularized ECM impacts macrophage polarization. They found that a three-dimensional lymph node extracellular matrix was more effective at promoting an anti-inflammatory macrophage phenotype along with enhanced macrophage elongation and phagocytic capacity compared to a two-dimensional ECM [79]. In an in vivo model of volumetric muscle loss, mice implanted with the 3D lymph node ECM showed increased levels of CD206^+^ macrophage recruitment to the site of injury 3 and 10 days postinjury, compared to control groups. This was reflected in H&E staining that showed lower levels of fibrosis and adipogenesis as compared to controls. Mice with the 3D lymph node ECM implant had improved gait analysis, indicating improved muscle function and coordination (Fig. 2a).

While decellularized ECM alone can promote an anti-inflammatory response, this can be enhanced in the presence of injected cytokines to drive local immune modulation. In the context of cartilage regeneration, decellularized cartilage matrix polarized macrophages towards a reparative phenotype, which in turn drove mesenchymal stem cell infiltration and chondrogenic differentiation in vitro. When injected into osteochondral defects in mice, the scaffold itself was able to increase the number of M2-like macrophages in the articular space. This value increased when the scaffold was delivered in combination with injections of IL-4, a pro-chondrogenic cytokine, leading to cartilage regeneration 4 and 8 weeks postinjury [80].

#### Biophysical Cues: Adaptive Immune Response

Beyond the initial innate immune response, native acellular ECM can drive downstream adaptive immune modulation as well. Anderson et al. designed an acellular adipose ECM for soft tissue reconstruction. Processing of the ECM was designed to retain biophysical cues while clearing lipids that can induce inflammation in the site of implantation. This ECM instigated an IL-4 driven M2-like phenotype, as well as elevated levels of eosinophil and helper T cell infiltration in a mouse model of muscle loss [81]. Notably, this study showed high translational promise as an acellular, off-the-shelf therapeutic, and demonstrating tolerability in small and large animal models as well as in humans. The therapeutic has progressed to Phase 1 clinical trials where reparative immune cell profiles were observed to be similar to those seen in mouse models (Fig. 2b). These implants showed potential for long-term tissue remodeling and regeneration in humans, but further analysis is needed to demonstrate the difference in immune cell response for allogeneic versus xenographic ECM origins.

Quantum dots (QDs) have been traditionally used as bio-imaging agents; however, their high surface-to-volume ratio and surface-bound reactive groups make them attractive candidates for applications in drug delivery, tissue engineering, and regenerative medicine. To this effect, titanium carbide MXene quantum dots were shown to downregulate effector T cells and promote the expansion of regulatory T cells in vitro [82]. Preliminary data shows that culturing cardiomyocytes on the MXene QD hydrogel scaffold leads to better transmission of electrical signals, illustrating the potential in vivo effect that this platform can have. While further investigation remains as to how MXene QD hydrogels influence the innate immune system and contribute to cardiac regeneration, this study merges mechanical material modification with electrical properties, a relatively unexplored aspect of material cues that holds promise.

### Chemical Modifications

Chemical modifications to biomaterials can enhance the base biomaterial’s tissue engineering properties through two main mechanisms. The first is changes to the fabrication process to chemically alter the structure of the biomaterial in order to promote a specific immune response. Additionally, chemical modifications can also encompass adding metal ions and biologics to the surface through different physicochemical reactions, such as layer-by-layer deposition or chemical conjugation to the scaffold [83]. These surface modifications enable ratiometric control of ligands on the surface, driving localized immune modulation at the interface of the material.

#### Chemical Modifications: Innate Immune Response

Variation in biomaterial fabrication methods can enhance the biomaterial’s effect on the immune system and at driving regeneration. Witherel et al. created a collagen scaffold cross-linked with various chemicals to identify which chemical modifications induced anti-inflammatory versus inflammatory phenotypes [84]. They found that chemical cross-linkers that induced temporary amide bond formations in the collagen gel were more likely to produce an anti-inflammatory phenotype in macrophages compared to cross-linkers that permanently altered collagen scaffold chemistry. Similarly, by varying the degree of degree of methyl esterification of pectin, Hu et al. was able to show that low levels of esterification on pectin alginate microcapsules led to decrease of Toll-like receptor 2-mediated immune activation in macrophages. This led to survival of long-term survival of islet-xenografts in a diabetes rat model [85]. Chemical modifications of biomaterials can also impact neutrophil behavior. Modification of a hydroxyapatite gelatin scaffold with strontium was shown to alter neutrophil-driven macrophage polarization to enhance angiogenesis in vitro and in vivo [86]. These studies highlight the importance of thoroughly characterized fabrication processes, as each component of a biomaterial has the potential to influence the microenvironment towards reparative or inflammatory.

**Fig. 3:**
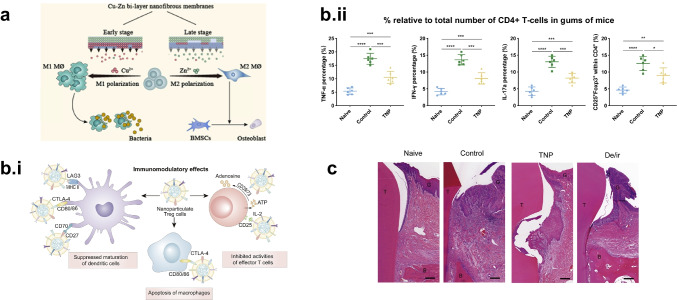
Chemical modification. (**a**) A brief schematic of the dual-temporal nanofibrous membrane used for fracture healing. An initial burst of copper modulates macrophages to an M1 state to encourage clearance of any potential residual bacteria in the fracture, while the secondary zinc release encourages M2 polarization and osteoblast differentiation for fracture healing. Adapted from Ref. [92] (**b.i**), PLGA nanoparticles coated with regulatory T cell membranes were able to modulate inflammatory actions of dendritic cells, macrophages, and effector T cells. (**b.ii**) In a mouse model of periodontis, after 15 days of treatment, the fraction of CD4.+ T cells produced inflammatory cytokines such as TNF⍺, IFN-ɣ, and IL-17a, as well as regulatory T cells. (**c**) H&E staining of mandibular ligature in a canine model of periodontist with B (bone area), G (gingiva), and T (tooth) areas were annotated. TNPs outperformed both the PBS group as well as the conventional treatment group (De/ir) — showing overall less ulcerous lesion formation and less attachment loss in the periodontal tissue. Adapted from Ref. [97]

Surface chemical modifications have been leveraged to mediate macrophage behavior for improved bone repair [87–89]. The Chu group and collaborators have used the biomaterial polyetheretherketone (PEEK) extensively for dental and orthopedic applications. PEEK has desirable properties for dental and orthopedic composites, which can be enhanced through layer-by-layer modifications that enhance bioactivity and alleviate the inflammatory immune response. Copper-coated PEEK demonstrated faster bacterial clearance and reduced bacterial loads in vivo, leading to overall improved bone health via micro-computed tomography [90]. Zinc-coated PEEK led to heightened release of anti-inflammatory cytokines from macrophages, skewing macrophage populations towards an anti-inflammatory phenotype that secretes osteogenic cytokines, improving bone regeneration [91]. Copper and zinc surface modifications were applied to nanofibrous membranes, where temporal control of copper and zinc presentation was able to sequentially shift macrophages from an inflammatory to anti-inflammatory state, promoting both an anti-microbial and osteogenic environment (Fig. 3a) [92].

#### Chemical Modifications: Adaptive Immune Response

Various labs have looked at how chemical modifications of biomaterial building blocks during fabrication can drive divergent regeneration responses in the adaptive immune system. The Segura lab has previously established and tested the use of microporous annealed particles (MAPs) as an injectable scaffold to encourage wound healing and tissue regeneration. By synthesizing a hydrogel made up of discrete microgel building blocks, the bulk lattice allows for efficient cell infiltration and tissue integration [93]. Furthermore, through incorporation of matrix metalloprotease-degradable peptides into MAPs, Griffin et al. were able to characterize the effect of peptide chirality on the adaptive immune response to this platform. In the context of dermal wound healing, they found that MAPs functionalized with D-enantiomer peptides specifically drove a Th2 response. An adaptive immune response was critical to wound healing, as mice without an adaptive immune system were not able to recapitulate wound the same wound healing as immune-competent mice [94]. Chemical modifications can also enhance ECM biocompatibility, injectability, and degradability. By modifying skeletal muscle ECM with hyaluronic acid, Estrellas et al. was able to enhance muscle regeneration in mice. Upon implantation, this modified scaffold elicited an M2-like response and higher regulatory T cell infiltration [95].

Surface modifications of biomaterials can enable localized presentation of biologics that otherwise have short half-lives and deleterious off-target effects when administered systemically. To this effect, PD-L1 checkpoint inhibitors were conjugated to PEG microgels to regulate the immune response after island graft transplantation [96]. These grafts were able to promote a tolerogenic immune environment, with enhanced recruitment of regulatory T cells and improved graft acceptance.

In addition to influencing adaptive immune cells, surface modifications can also create particles that can mimic adaptive immune cells themselves. Li et al. fabricated and characterized a PLGA nanoparticle that was coated with a regulatory T-cell membrane to create a biomimetic nanoparticle (Fig. 3b.i). These particles were able to inhibit macrophage-osteoclast differentiation, suppress dendritic cell activation, and suppress activation and proliferation of CD3^+^ T cells in vitro. In mouse and preclinical canine models of periodontis, the authors injected regulatory T-cell membrane-coated nanoparticles into the site of inflammation and observed decreased levels of inflammatory cytokines and regulatory T cells (Fig. 3b.ii). These particles were also able to mitigate alveolar bone resorption, inhibit ulcerous lesion formation, and lower clinical attachment loss in the periodontal tissue [97].

### Drug Delivery

Beyond providing chemical and biophysical cues through material properties to facilitate immune-mediated repair, biomaterials can serve as a platform for localized delivery of drugs and biologics. By tuning the properties of the biomaterial, therapeutic release can be altered in a temporal manner [98, 99]. Furthermore, delivery with biomaterials eliminates the challenges associated with systemic delivery of immunomodulatory cargos, overcoming rapid clearance, short half-lives, and deleterious off-target effects [100].

#### Drug Delivery: Innate Immune Response

Many different material platforms have been used to elute cytokines, exosomes, or drugs to tune macrophage activity and subsequently downregulate the inflammatory response to promote tissue regeneration. Local delivery of a cytokine cocktail containing IL-4 and CSF-1 in alginate gels demonstrated promise in murine models, promoting reparative CD206^+^ macrophage phenotypes that led to accelerated wound closures and stabilizing cardiac function in ischemic skin and myocardium injuries, respectively [101]. IL-33 releasing hydrogels have been used to reduce pro-inflammatory macrophage populations, mediating their fatty acid uptake to downregulate inducible nitric oxide synthase (iNOS) expression and drive a reparative phenotype. This treatment was shown to have a local effect and reduced graft rejection in a murine model a murine model of allografted heart transplants. IL-33 modulation was also found in clinical samples of cardiac transplants, where deficiencies in IL-33 correlated strongly with rejection, highlighting the potential for this therapeutic to serve as a prophylactic chronic rejection treatment [102]. Similarly, islet cell transplantation can be augmented by hydrogels that release a combination of IL-4 and dexamethasone to promote an M2, anti-inflammatory macrophage population in local environment, and improve islet cell survival for diabetes treatment [103].

To promote the successful integration of vascular grafts and limit neointimal hyperplasia and subsequent restenosis, our group alongside clinical collaborators has utilized poly(lactic-co-glycolic acid) (PLGA) wraps for unidirectional release of the anti-inflammatory lipid mediator Resolvin D1. Localized application of wraps at the site of the graft attenuated neointimal hyperplasia and reduced macrophage infiltration into the neointima in rabbit models [104]. Importantly, use of these wraps did not lead to increased postoperative complications. With PLGA as an approved drug delivery device by the Food and Drug Administration, these vascular wraps hold promise for long-term translatability.

The usage of exosomes has also gained popularity in recent years, with exosomes derived from mesenchymal stem cells demonstrating immunomodulatory effects on a variety of cells [105, 106]. Xin et al. overcame the difficulties of umbilical cord-derived mesenchymal stem cell therapy for intrauterine adhesions by creating a collagen scaffold embedded with stem cell-derived exosomes. This matrix was able to confer the same benefits as stem cell therapy and facilitate endometrial regeneration via M2 macrophage polarization in vitro and in vivo [105]. Further investigation into exosome cargo and its downstream influence on macrophages need to be performed in order to determine the molecular drivers of immune cell phenotype regulation.

Biomaterials can also augment drug delivery in traditionally hard-to-target areas, such as the brain. A hyaluronic acid hydrogel containing vascular endothelial growth factor (VEGF) injected into the stroke cavity promoted neural differentiation and re-vascularization [107]. Microglia, macrophage-like cells of the central nervous system, were less activated in response to the VEGF hydrogel, promoting an anti-inflammatory, regenerative phenotype.

#### Drug Delivery: Adaptive Immune Response

**Fig. 4:**
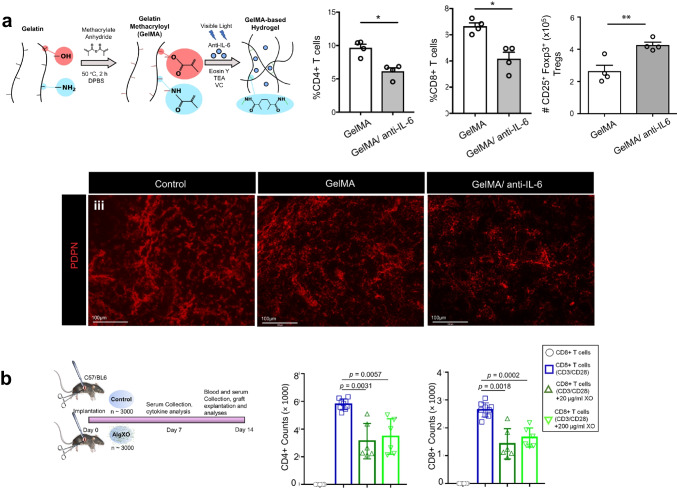
Drug delivery. (**a**) In a skin transplant mouse model, an anti-IL6 eluting gelatin methacryloyl–based hydrogel reduced infiltration of CD4 and CD8 positive T-cells in the skin and higher levels of regulatory T cells in the draining lymph nodes. The draining lymph nodes also had lower levels of fibrosis, as shown by the stain of podoplanin (PDPN), an extracellular matrix protein. Adapted from Ref. [108]. (**b**) Exosome-loaded alginate microcapsules were fabricated to alleviate the local immune response after islet transplantation in diabetic mouse models. Purified CD3+ T cells were isolated and incubated with exosome-loaded alginate microcapsules. Addition of alginate microcapsules reduced the fraction of CD4+ and CD8.+ T cells, illustrated suppressed T cell proliferation. Adapted from Ref. [106]

Delivery of cytokines or cytokine inhibitors in biomaterial scaffolds have shown to be an effective approach to affect T cell populations, extending the survival of engraftment treatments [108, 109]. These scaffolds serve a two-fold function — providing sustained local release of therapeutics and structural support for the engrafted cells or tissues to interface with the host tissue. Anti-IL-6 delivered in gelatin methacrylate scaffolds blocked the inflammatory action of IL-6, reducing immune cell infiltration and inflammatory T cell populations in a skin graft model. The authors also observed a local expansion of Tregs and reduction in fibrosis surrounding the graft, overall prolonging graft survival (Fig. 4a) [108]. Similarly, delivery of IL-33 through PLGA scaffolds promoted local Treg populations and extended transplanted islet survival, reversing diabetes in a mouse model. Interestingly, IL-33-loaded scaffolds also delayed engraftment of islet cells to achieve normoglycemia [109].

In addition to the delivery of cytokines, other therapeutics have been explored for immunomodulatory drug delivery. Nanoparticle-based delivery of bilirubin extended the circulation time of bilirubin and suppressed type 2 inflammation through reduction of associated T cell populations, attenuating asthma symptoms and inflammation [110]. Co-delivery of exosomes and islet cells encapsulated in alginate was able to significantly extend islet functionality and maintain normoglycemia compared to implanted constructs without exosomes, downregulating inflammatory cytokine production, and suppressing T cell proliferation (Fig. 4b) [106].

While many immunomodulatory therapies drive anti-inflammatory responses and quiescence of aberrant inflammation, biomaterial strategies to promote inflammatory responses are still relevant in specific circumstances. Inflammatory cytokines play a key role in mediating the resolution of inflammation, driving a variety of cells to produce anti-inflammatory signals [111]. One such example is IFN-ɣ activating mesenchymal stem cells to produce immune modulating cytokines and prevent fibrotic progression, where IFN-ɣ is in fact crucial to the immunomodulatory effect of mesenchymal stem cells [112]. Incorporation of IFN-ɣ into PEG hydrogels encapsulating human mesenchymal stem cells (hMSCs) has proven to be an elegant approach to eliminate the need for ex vivo stimulation and timing. Injection of these systems led to increased production of immunomodulatory cytokines and decreased T cell proliferation, leading to repair of colonic wound models in mice [113]. As seen in these studies, disease-specific repair can be achieved through biomaterial design where therapeutic selection will be dependent on the repair approach as well as the target tissue.

### Sequestration of Endogenous Signaling Factors

In lieu of loading and releasing exogenous therapeutic molecules, biomaterials can also be designed to sequester and retain endogenous repair factors produced by the body. Such scaffolds can provide some additional biomimicry of the extracellular matrix, providing structure for cellular adhesion and migration while also presenting reparative factors. Creating this “sink” of growth factors and chemokines can be achieved through various approaches discussed in depth elsewhere [114, 115], including the incorporation of aptamers [116, 117], conjugation of antibodies [118], and addition of extracellular matrix components [119]. Extending the half-life of these therapeutic factors in damaged tissue environments can skew responding immune cells to drive reparative mechanisms and accelerate repair.

#### Sequestration: Innate Immune Response

**Fig. 5:**
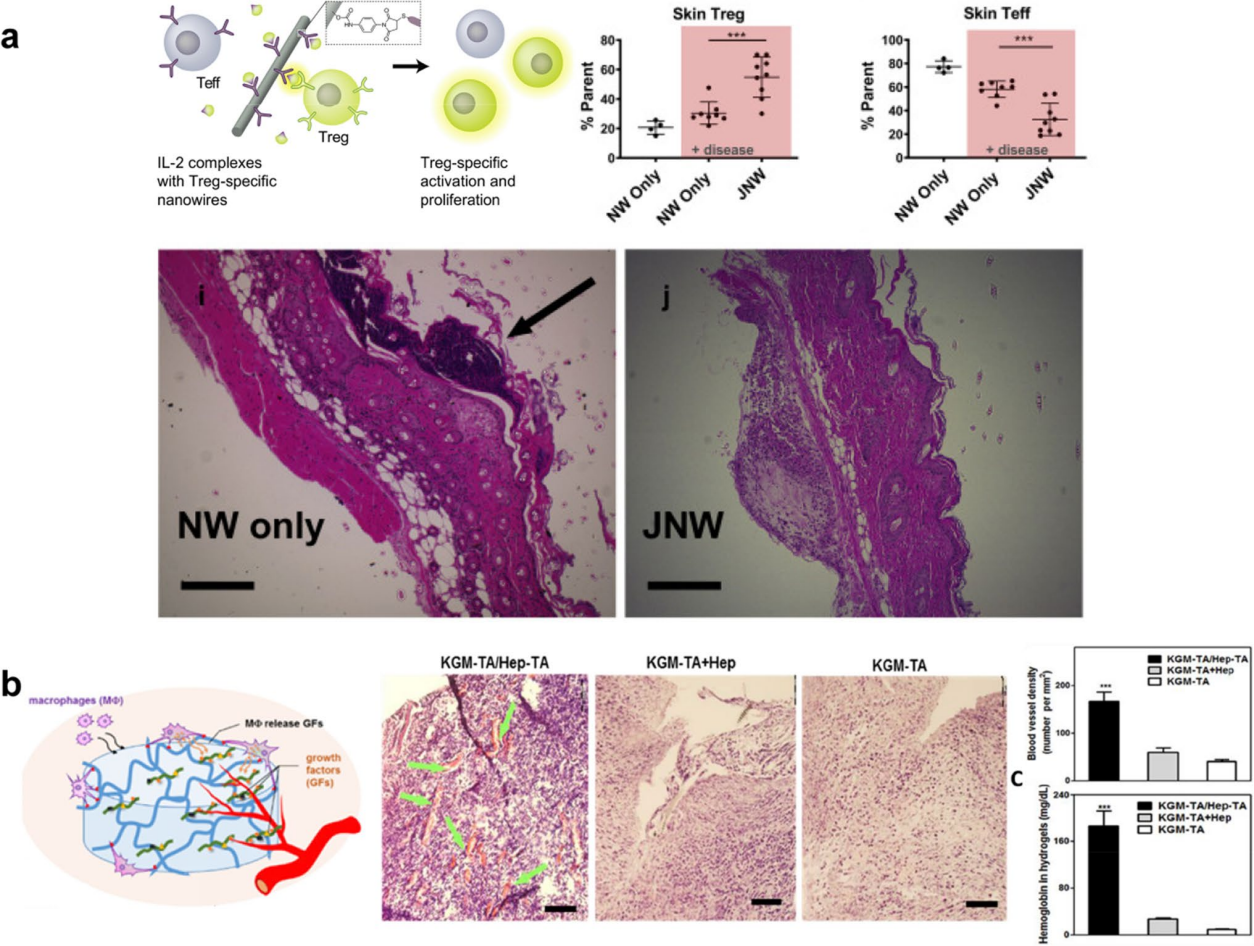
Sequestration. (**a**) Design of an injectable cytokine nanowire scaffold conjugated to anti-IL2 antibody to recruit endogenous IL-2 into the tissue and drive a localized regulatory T cell response. In a transgenic mouse model of autoimmune disorders, treatment with this biomaterial led to higher levels of regulatory T cells and lower levels of effector T cells. The representative H&E stains show that the thickness of skin plaque decreased in the treatment group with anti-IL2 antibody conjugated to the nanowires compared to the nanowires themselves, illustrating the positive effects of a localized regulatory T cell response. Adapted from Ref. [121]. (**b**) A hydrogel made of heparin and glucomannan serves as a sink for endogenous growth factors that can promote vascularization, as seen in the H&E staining of the KMG-TA/Hep-TA group. This hydrogel led to increased blood vessel density and hemoglobin levels when implanted subcutaneously in mice. Adapted from Ref. [118]

Vascularization has posed a large challenge in advancing the field of tissue engineering to provide conduits for nutrient transport and circulating cells to injured tissue. Modulation of macrophage phenotypes has shown that many different activation states contribute to vascularization, each producing different growth factors [37]. Crucially, VEGF plays a key role in endothelial tube formation and branching, with higher densities promoting vessel growth and infiltration [120]. Feng and colleagues incorporated heparin, a glycosaminoglycan well known for its abilities to bind and retain VEGF, into polysaccharide-based polymer scaffolds, where they demonstrated that this scaffold activated macrophages in an M2-like manner and increased their production of VEGF. Heparin sequestration of this macrophage produced VEGF and led to increased vessel formation within the injury site (Fig. 5a) [121]. This approach to induce immune cell states and sequester key growth factors secreted all within one material platform presents an elegant design that could be further explored.

#### Sequestration: Adaptive Immune Response

Interleukin-2 (IL-2) is a key cytokine for the activation and proliferation of T cells [122]. Selective activation of T cell subpopulations can be accomplished through administration of IL-2 complexed with antibodies that preferentially select for regulatory or effector T cells [123–125]. Recent work from the Desai lab has harnessed these anti-IL-2 antibodies to sequester endogenous IL-2 to locally modulate T cell populations in diseased tissue environments. Conjugation of antibodies to polycaprolactone nanowires allowed for retention of IL-2 at the site of injection, enhancing tissue-specific T cell amplification while preventing off-target effects in draining lymph nodes [126]. This approach was subsequently applied to a murine model of psoriasis, an autoimmune disease characterized by lesions and plaques of the skin. Injection of nanowires conjugated with Treg-promoting JES6-1 anti-IL-2 antibodies selectively increased local Treg populations. Expansion of this suppressive T cell group led to a decrease in inflammatory cytokines and reduction in plaque thickness, indicating improvements in tissue remodeling (Fig. 5b) [118]. Overall, the use of biomaterials to harness endogenous signaling molecules to induce localized changes in tissue-specific immune populations and promote regenerative engineering holds great promise.

## Conclusion and Future Outlook

The immune system plays a critical role in the reparative processes following injury and disease, and thus presents a clear target for tissue engineering strategies. Material-based strategies have demonstrated the ability to modulate the immune system towards favorable responses, and we have now seen application of these strategies to promote regenerative outcomes. Through the rational design of biomaterial-based systems, an increasing number of biomaterial approaches have presented a variety of mechanisms to deliver immunomodulatory cues in a localized manner. These platforms modulate local immune populations through innate material properties, bioactive surface modifications, drug delivery, and sequestration of endogenous factors, creating favorable microenvironments for repair.

Ultimately, to accomplish the goals of tissue engineering within this paradigm, immunomodulatory biomaterials must be translatable to the clinic. Very few biomaterial-based therapies have made it to clinical trials thus far, and even fewer with immunomodulatory components. To this extent, there are still many considerations that must be better understood. The long-term compatibility of these immunomodulatory biomaterials must be further assessed, as prolonged immune altering effects beyond repair may lead to disruption of homeostasis. Patient-to-patient immune system variability is especially important to understand for treatment effectiveness [127], as are the differences in immune systems between pre-clinical models and human clinical trials. Better understanding of immune-material interactions will help address these considerations and guide translation of materials into the clinic. Representative in vitro models of injury microenvironments or organoid cultures offer promise as approaches to decipher specific immune cell-material behaviors, while further profiling of animal models provides a more expansive view of immune responses.

We are still faced with many of the same questions described half a decade ago in understanding the complexity of interactions surrounding immune-mediated repair [11]. Our understanding of immune cell-material interactions has significantly improved, driven by multi-omic analyses at the single cell level [128–131]. Further analyses would advance our understanding of not only lesser studied immune cells (e.g., neutrophils, DCs, B cells) but also the role immune populations play in various disease spaces as well as the molecular interactions that lead to changes to immune cells. By deciphering the heterogeneity of functional and phenotypic changes in immune cells in response to biomaterials and unraveling the web of associated cellular communications, a clear design language for biomaterials will emerge. Further immune profiling to understand these cell-material interactions will help guide the design of the next generation of biomaterial platforms.

### *Citation Diversity Statement, Adapted from Ref. *[132]

Recent work in several fields of science has identified a bias in citation practices such that papers from women and other minorities are under-cited relative to the number of such papers in the field [133–136]. Here, we sought to proactively consider choosing references that reflect the diversity of the field in thought, form of contribution, gender, and other factors. Via manual curation, we determined predicted gender and predicted ethnicity of both the first and last authors of the references cited in this paper. By this measure (excluding self-citations), our references contain 21% woman (first author)/woman (last author), 11% man/woman, 28% woman/man, and 40% man/man. This method is limited in that (a) names, pronouns, and social media profiles used to construct the databases may not, in every case, be indicative of gender identity and (b) it cannot account for intersex, non-binary, or transgender people. Predicted racial ethnicity of our references contain 31% author of color (first)/author of color (last), 7% white author/author of color, 24% author of color/white author, and 38% white author/white author. This method is limited in that names may not be indicative of racial/ethnic identity, and (b) it cannot account for indigenous and mixed-race authors, or those who may face differential biases due to the ambiguous racialization or ethnicization of their names. We look forward to future work that could help us to better understand how to support equitable practices in science.
